# Galacto-Oligosaccharides Modulate the Juvenile Gut Microbiome and Innate Immunity To Improve Broiler Chicken Performance

**DOI:** 10.1128/mSystems.00827-19

**Published:** 2020-01-14

**Authors:** Philip J. Richards, Geraldine M. Flaujac Lafontaine, Phillippa L. Connerton, Lu Liang, Karishma Asiani, Neville M. Fish, Ian F. Connerton

**Affiliations:** a Division of Microbiology, Brewing and Biotechnology, School of Biosciences, University of Nottingham, Loughborough, Leicestershire, United Kingdom; b Dairy Crest Ltd., Dairy Crest Innovation Centre, Harper Adams University, Edgmond, Newport, United Kingdom; University of Southampton

**Keywords:** prebiotic, galacto-oligosaccharides, chicken, microbiome, gut health, synbiotic, innate immunity, IL-17A

## Abstract

Improvements in growth performance and health are key goals in broiler chicken production. Inclusion of prebiotic galacto-oligosaccharides (GOS) in broiler feed enhanced the growth rate and feed conversion of chickens relative to those obtained with a calorie-matched control diet. Comparison of the cecal microbiota identified key differences in abundances of *Lactobacillus* spp. Increased levels of Lactobacillus johnsonii in GOS-fed juvenile birds at the expense of Lactobacillus crispatus were linked to improved performance (growth rate and market weight). Investigation of the innate immune responses highlighted increases of ileal and cecal interleukin-17A (IL-17A) gene expression counterposed to a decrease in IL-10. Quantification of the autochthonous *Lactobacillus* spp. revealed a correlation between bird performance and L. johnsonii abundance. Shifts in the cecal populations of key *Lactobacillus* spp. of juvenile birds primed intestinal innate immunity without harmful pathogen challenge.

**IMPORTANCE** Improvements in the growth rate of broiler chickens can be achieved through dietary manipulation of the naturally occurring bacterial populations while mitigating the withdrawal of antibiotic growth promoters. Prebiotic galacto-oligosaccharides (GOS) are manufactured as a by-product of dairy cheese production and can be incorporated into the diets of juvenile chickens to improve their health and performance. This study investigated the key mechanisms behind this progression and pinpointed L. johnsonii as a key species that facilitates the enhancements in growth rate and gut health. The study identified the relationships between the GOS diet, L. johnsonii intestinal populations, and cytokine immune effectors to improve growth.

## Abstract (precis)

Improvements in the growth rate of broiler chickens can be achieved through dietary manipulation of the naturally occurring bacterial populations while mitigating the withdrawal of antibiotic growth promoters. Prebiotic galacto-oligosaccharides (GOS) are manufactured as a by-product of dairy cheese production and can be incorporated into the diets of juvenile chickens to improve their health and performance. This study investigated the key mechanisms behind this progression and pinpointed L. johnsonii as a key species that facilitates the enhancements in growth rate and gut health. The study identified the relationships between the GOS diet, L. johnsonii intestinal populations, and cytokine immune effectors to improve growth.

## INTRODUCTION

The production of poultry for both meat and eggs has been increasing rapidly throughout the world (1), and the global poultry sector is expected to continue to grow as a result of growing population, rising income, and urbanization (2). In this context, animal performance and feed conversion efficiency of fast-growing birds are decisive to the economic profitability of poultry meat production. Broiler chicken production is more sustainable and has a relatively lower environmental impact than other meat-based protein production (3). There have been massive increases in the growth rate and feed efficiency of broiler chickens since the 1940s, achieved largely through selective breeding and feed optimization (4). It is generally recognized that increases in performance are slowing as the advances made possible through these approaches are reaching their biological limit (5). The inclusion of antimicrobial growth promoters (AGPs), a practice banned in the European Union (EU) since 2006, is another way in which gains in productivity have been realized. The EU ban was imposed due to increasing concerns regarding the development of antimicrobial resistance and the transference of antibiotic resistance genes from animal to human microbiota (6). Although antimicrobials are still widely used, there have been reductions in the therapeutic use of antimicrobials in poultry production, which have led to an increase in intestinal health problems (7). To mitigate the effect of antimicrobial reduction a variety of strategies has been evaluated (8). These include the addition of dietary prebiotic (9, 10), the use of phytobiotic dietary additives (11), the incorporation of beneficial enzymes in poultry feed (12), and the administration of live probiotic bacteria in various combinations of the above (13). Recent developments in sequencing technologies have led to a greater understanding of the mechanisms and effects of these treatments on the gut microbiota and the interaction with host-related functions involved in intestinal health (14, 15). It has been proposed that further improvements to broiler performance could be sought through deliberate cultivation of a beneficial gut microbiota in early development (7, 16). These bacteria are preferably autochthonous and mutualists in association with each other and their host.

Galacto-oligosaccharides (GOS) are nondigestible carbohydrates that have been shown to promote beneficial autochthonous bacteria, such as *Bifidobacterium*, *Bacteroides*, and *Lactobacillus*. GOS are synthesized from lactose by β-galactosidase-catalyzed transglycosylation to create molecules of differing lengths and linkage types (17, 18). Several studies have reported that GOS can improve the performance of poultry and produce profound differences in desirable bacterial groups inhabiting the gut (19, 20).

Maintenance and enhancement of gut health are essential for the welfare and productivity of animals (21). In addition to nutrient digestion and absorption, the intestinal mucosa constitutes a physical and immunological protective barrier for the integrity of the intestinal tract (22). Mutualistic commensals with immunomodulotary effects (autobionts) affect the development and function of various immune cell populations, such as regulatory T cells (Tregs), Th17 T-helper cells, IgA-secreting plasma cells, natural killer (NK) cells, macrophages, dentritic cells (DCs), and innate lymphoid cells (ILCs) (23). Interleukin-17-producing CD4^+^ T lymphocytes (Th17 cells) contribute to host defense against pathogens and maturation of the immune response at an early age. Regulatory T cells play critical roles in immune suppression (24, 25), and thus, optimum health is achieved through a balanced regulation of expression between Th17 cells and Tregs.

There is little systematic information regarding the interaction between prebiotic diet, performance, structure of gut microbiota, and host gene expression in poultry. In this study, the impact of a GOS diet was assessed in broiler chickens by comparing a cohort fed a control diet and a cohort fed a GOS diet from day of hatch until 35 days of age (da), corresponding to a typical commercial farm rearing period. Ancillary dietary trials were carried out to confirm the reproducibility of the beneficial effects. The innate immune responses to the two diets were assessed in ileal and cecal tissue biopsy specimens by quantification of the relative expression of cytokine and chemokine gene transcription. Analysis of metagenomic profiles of GOS-fed birds enabled the identification and isolation of autochthonous synbiotic organisms. Characterization of these isolates allowed an in-depth analysis of the effect of the GOS diet and synbiotic species abundance on bird performance and gut health.

## RESULTS

### GOS supplementation improves the growth performance of broiler chickens.

**FIG 1:**
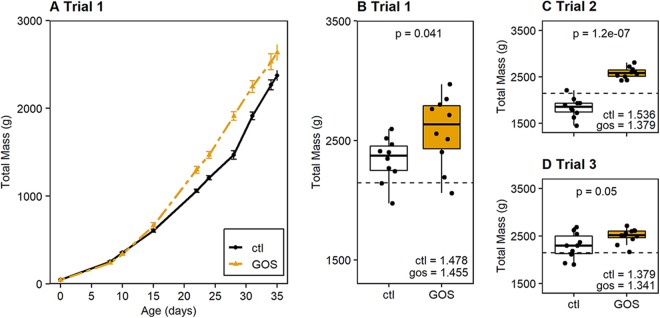
Galacto-oligosaccharide diet improves the growth performance of broiler chickens. (A) GOS diet trial 1 comparing median body weight of birds fed the GOS diet with that of birds fed the control (ctl) diet. Data presented are for the mass observations made for the 10 birds from each cohort that remained at 35 days and hence were recorded throughout the trial. The contemporary male Ross 308 broiler chicken performance objective weight progression (96) is indicated by the gray dashed line. Panel B shows a box-and-whisker plot of the bird weights for trial 1 at 35 da. Panels C and D show box-and-whisker plots of the bird weights at 35 da for ancillary GOS diet trials 2 and 3 to demonstrate that the birds on the GOS diet consistently achieved greater body weight at 35 da than birds provided with a calorie-matched control diet. For reference, the contemporary male Ross 308 performance objective at 35 da is indicated by a horizontal dashed black line in each panel (96). Comparisons were made of mean weights using Student’s *t* test, with the corresponding *P* values reported above the diet pairs and the corresponding cumulative feed conversion ratios (FCR) indicated in the bottom right-hand side of each trial panel.

Chickens fed a galacto-oligosaccharide (GOS)-supplemented diet performed better than those fed an isocaloric control diet (Fig. 1A). An increase in growth rate was apparent for the GOS-fed birds, which exhibited an increase in the mean live weight of 87.7 g/day, compared to 76.3 g/day for the control birds, calculated at between 8 and 35 days of age (da) (*P = *0.012). Correspondingly, the mass of GOS-fed birds in trial 1 was greater than that of control birds at the slaughter age of 35 days (GOS-fed birds, 2,582 g; control birds, 2,336 g; *P = *0.041 [Fig. 1B]). Two ancillary trials were carried out to demonstrate whether after removing the prebiotic once the microbiota was established on the GOS feed the beneficial effects on performance could be reproduced in mature birds. In these trials, the birds were either housed in individual cages (trial 2) or cohoused in 10 pens containing 3 birds (trial 3). The enhanced performance on the GOS-supplemented diet was evidenced by greater masses at 35 da in trial 2 (GOS-fed birds, 2,584 g; control birds, 1,838 g; *P < *0.001 [Fig. 1C]) and trial 3 (GOS-fed birds, 2,501 g; control birds, 2,291 g; *P = *0.057 [Fig. 1D]). Although trial 3 marginally failed to meet significance, the trend remained the same, with the GOS diet producing beneficial effects on growth when the birds were reared in pens. The feed conversion ratios (FCR) varied between trials but were reduced for birds on the GOS diets compared to the corresponding control groups (Fig. 1B to D). The zootechnical performance data for all trials are summarized in Table S1 in the supplemental material; they repeatedly show significantly greater weights for the GOS-fed juvenile birds over those on the control diet reared under similar circumstances at 35 da.

10.1128/mSystems.00827-19.3 TABLE S1 Growth performance of broiler chickens raised on GOS-supplemented feed. Download Table S1, DOCX file, 0.03 MB. Copyright © 2020 Richards et al. 2020 Richards et al. This content is distributed under the terms of the Creative Commons Attribution 4.0 International license.

### *Lactobacillus* spp. distinguish microbial communities colonizing the ceca of broiler chickens on GOS-supplemented diets.

Cecal bacterial communities were surveyed using 16S rRNA gene sequences. Analysis of trial 1 showed that the α-diversities of the cecal microbiota were not significantly different between GOS and control diet cohorts sampled at 8, 15, 22, and 35 da (inverse Simpson index, *P* ≥ 0.295; Shannon diversity, *P* ≥ 0.187 [Fig. S1A and B]). Community richness (Chao) was not significantly different throughout the trial (*P* ≥ 0.101 [Fig. S1C]). Communities of cecal bacteria colonizing birds on control and GOS diets could not be distinguished on the basis of Bray-Curtis dissimilarity at any age (*P* > Bonferroni correction for pairwise error [Fig. S1D]).

10.1128/mSystems.00827-19.1 FIG S1 Diversity of the cecal microbiota on GOS and control diets. Panels A to C show indices of α-diversity of cecal bacterial populations for broiler chickens on GOS (gold) and control (black/white) diets sampled through the rearing period (8 to 35 da). Panel D shows NMDS plots of Bray-Curtis dissimilarity indices for all birds at all sample days. Age-matched cecal microbial communities could not be distinguished at any time point (pairwise error rate [Bonferroni] > 0.0017). Download FIG S1, TIF file, 2.9 MB. Copyright © 2020 Richards et al. 2020 Richards et al. This content is distributed under the terms of the Creative Commons Attribution 4.0 International license.

**FIG 2:**
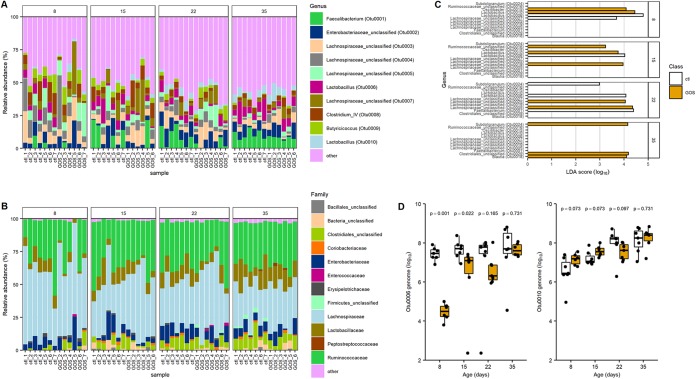
Dietary GOS shifts the abundance of specific taxa. Panels A and B show stacked bar charts indicating the relative abundance to OTU (A) and family (B) levels of the cecal microbiota of birds fed GOS and control diets. In three cases, the sequence data sets did not meet pre- or postprocessing quality thresholds and were removed from the analysis (GOS samples at 8, 15, and 35 da). Panel C shows the differential bacterial species identified in cecal contents from birds fed the control diet and GOS diet analyzed using linear discriminant analysis effect size (LEfSe). Prior to analysis, age-matched communities were filtered to include only OTUs at ≥1% relative abundance. The linear discriminant analysis (LDA) threshold was set at 2, and the *P* value threshold was set at 0.05. Panel D shows box-and-whisker plots of the genome copy numbers of OTU0006 and OTU0010 per gram of cecal content determined using qPCR. Data are reported for genome numbers identified in birds fed GOS and control diets. Significant differences are indicated by *P* values above the sample pairs.

The top 10 operational taxonomic units (OTUs) with the greatest relative abundances are shown in Fig. 2A for all the birds sampled at each time point. Figure 2B shows family level taxonomy to confirm the similarity between the cecal bacterial communities of birds fed control and GOS diets. However, few OTUs were discriminative between the control and GOS diets (Fig. 2C). Two OTUs, OTU0006 and OTU0010, identified as *Lactobacillus* spp., were discriminatory of the different diets in the early rearing period up to 15 da (OTU0006, *P* ≤ 0.01; OTU0010, *P* ≤ 0.022). Reads representing these OTUs accounted for 84.5% of those assigned to the *Lactobacillales* order and 86.1% assigned to the *Lactobacillaceae* family (Fig. 2B). The third major *Lactobacillus* species is represented by OTU0017, which together with OTU0006 and OTU0010 constitutes 99.95% of the *Lactobacillaceae*. Organisms exhibiting 16S rRNA gene V4 region sequence identity with the differential OTUs identified from 16S rRNA community analysis were isolated from MRS culture media. The genomic DNA sequences of these isolates were assembled from data generated on Illumina MiSeq and PacBio RSII platforms. Two representative isolates were designated L. crispatus DC21.1 (OTU0006) and L. johnsonii DC22.2 (OTU0010) based on whole-genome alignments with type strains available in public nucleotide sequence databases.

Quantitative PCR assays were developed to measure the absolute abundances of L. johnsonii (OTU0010) and L. crispatus (OTU0006) within the gut microbiota. Oligonucleotide primers were designed on the *groEL* gene sequences, as they have been frequently used to discriminate between *Bifidobacterium* strains with a high degree of sequence similarity (26, 27). Once validated, using spiked cecal samples, the technique was used to enumerate L. johnsonii (OTU0010) and L. crispatus (OTU0006) organisms within the cecal contents of control and GOS-fed birds. The genome copies of each isolate were measured throughout the rearing period. The results of these analyses confirmed the relative abundance estimates from metagenomic data in trial 1 and demonstrated that the abundances of these two OTUs show positive and negative associations with the GOS diet compared to the control diet (Fig. 2C). Notably, the abundance of L. crispatus (OTU0006) in the GOS-fed birds at 8, 15, and 22 da was significantly lower than in control birds (*P *< 0.048), and conversely, the abundance of L. johnsonii (OTU0010) was significantly greater at 8 da in the GOS-fed birds (*P = *0.001).

### Characteristics of the *Lactobacillus* spp. distinguishing the cecal communities.

Summaries of the functional gene contents of the L. johnsonii DC22.2 and L. crispatus DC21.1 isolates with respect to GOS utilization and host colonization are presented in Table 1. L. johnsonii and L. crispatus have the capacity to colonize and compete in the host intestine with genes encoding multiple mucus binding proteins (28), fibronectin binding proteins (28), exopolysaccharide biosynthesis (29), and bile salt hydrolase (30). L. johnsonii carries the *apf* gene (aggregation promoting factor), which encodes a cell surface protein that has been assigned a role in cell adhesion (31). L. crispatus contains the *cbsA* gene, which encodes the structural protein that forms the S-layer (32, 33). L. johnsonii and L. crispatus both encode the bacteriocin helveticin J and multiple bacteriocin immunity factors.

**TABLE 1:** Summary of the functional gene contents of L. johnsonii and L. crispatus isolates related to GOS utilization and host colonization

Gene(s)	L. johnsonii locus tag E6A54_	L. crispatus locus tag E6A57_	Function	Reference
*lacS*	06610^*a*^	07265	Lactose permease	41
*lacA*	06605	07260	β-Galactosidase	41
*lacL/M*	06620	07275	β-Galactosidase	34
	06625	07280		
*lacE/F*		O7225	PTS lactose transporter	34
*lacG*			Phospho-β-galactosidase	34
*mucBP*	04955	06095	Adhesion	28
	08660^*a*^	07570		
	09625	08180		
		01350		
*fbpA*	04640	05230	Adhesion	28
*cbsA/B*		00840	S-layer	32
*apf1/2*	07620		Aggregation factor	31
*epsA–E*	05690–05730	08695–08755	Exopolysaccharide	29
Bacteriocin leader		09005	Bacteriocin	28
*hlv*	02750	00185	Helveticin J	28
		05290		
		07800		
		10185		
*bsh*	00415	08515	Bile salt hydrolase	30

a Internal stop codons present.

There are ostensibly two pathways to utilize GOS that rely upon the cellular transporters LacS (lactose permease) and LacE/F (lactose phosphotransferase system); LacS permease appears to be capable of transporting GOS with degrees of polymerization of 2 to 6 (DP2 to DP6), but the LacE/F phosphotransferase is confined to DP2 lactose (34). The genome sequence of L. johnsonii DC22.2 suggests that the isolate could be impaired in GOS utilization. The *lacS* permease gene has a stop codon at the 17th position, which would require that the protein be initiated from an internal AUG with the loss of the first 31 amino acids compared to the majority of database homologues. In contrast, L. crispatus DC21.1 retains functional *lacS* and *lacA* genes to facilitate the use of GOS.

**FIG 3:**
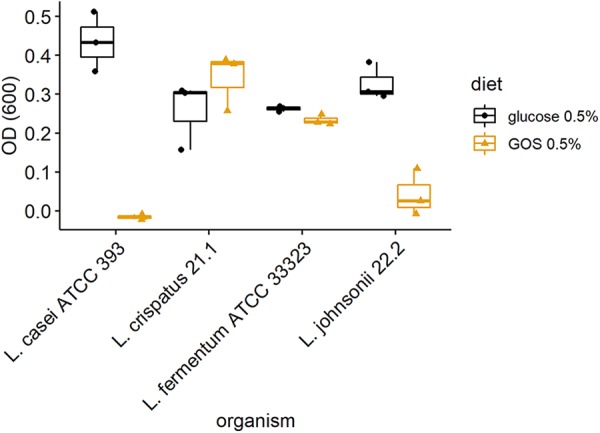
*In vitro* growth of lactobacilli on galacto-oligosaccharides. Utilization of DP2+ GOS by L. crispatus DC21.1 and L. johnsonii DC22.2 isolates compared with that by *Lactobacillus* type strains is shown. Anaerobic growth was recorded from OD_600_ measurements after incubation with basal medium that was subtracted from that obtained by incubation with basal medium plus DP2+ GOS or glucose as a carbon source.

To assess the ability of the L. crispatus and L. johnsonii isolates to utilize GOS in axenic culture, the organisms were cultured in MRS basal medium containing DP2+ GOS in the absence of monosaccharides. Cultures were incubated for 72 h under anaerobic conditions at 37°C in basal medium with either DP2+ GOS (0.5% [wt/vol]) or glucose (0.5% [wt/vol]) as a positive control or sterile water instead of the carbon source as negative control (blank). Figure 3 shows growth of the isolates and *Lactobacillus* type strains indicated by the measurements of optical density at 600 nm (OD_600_) corrected for the negative control. L. crispatus DC21.1 utilizes GOS, showing increased growth over that recorded for L. fermentum ATCC 33323 in a parallel culture. Under these conditions L. johnsonii does not efficiently use GOS, which is consistent with the putative gene content but at odds with the differential abundance observed in the ceca of GOS-fed birds.

### In-feed GOS effects on gut architecture.

Hematoxylin-eosin (H&E) stains of ileal histological sections did not exhibit any significant differences in heterophil infiltration or inflammatory characteristics between control and GOS-fed birds at any time in the experiment. The measurements of villus length and crypt depth indicate that GOS-fed juvenile birds at 15 da had longer villi (*P = *0.05) and deeper crypts (*P = *0.02) than birds on the control diet (Table 2). However, these differences did not result in a difference in the villus/crypt ratio. At 22 and 35 da, there were no significant morphometric differences recorded. Goblet cell densities of villi from control and GOS-fed birds were evaluated from periodic acid-Schiff (PAS)-stained (neutral mucin-producing) ileal sections (Table 2). Greater densities of goblet cells were observed from GOS-fed birds sampled throughout trial 1, with significant differences recorded at days 8 (*P = *0.04), 15 (*P = *0.002), and 22 (*P = *0.04).

**TABLE 2:** Ileal gut morphometrics: villus length, crypt depth, and goblet cell density

Diet or parameter and age (days)	Villus length (μm)	Crypt depth (μm)	Ratio villus/crypt	Goblet/mm^2^
Control diet				
8	475 ± 64	75 ± 7	6.3 ± 0.9	1,457 ± 347
15	615 ± 70	103 ± 11	6.0 ± 0.5	1,198 ± 140
22	747 ± 167	126 ± 20	5.9 ± 0.4	1,378 ± 290
35	731 ± 110	121 ± 17	6.0 ± 0.3	1,267 ± 243
GOS diet				
8	505 ± 63	79 ± 7	6.0 ± 0.8	1,707 ± 212
15	676 ± 93	113 ± 13	5.9 ± 0.5	1,450 ± 247
22	687 ± 118	118 ± 18	5.8 ± 0.3	1,616 ± 360
35	789 ± 162	126 ± 19	6.2 ± 0.6	1,394 ± 218
*P* value				
8	0.25	0.15	0.90	0.04
15	0.05	0.02	0.81	0.002
22	0.26	0.25	0.30	0.04
35	0.46	0.24	0.28	0.16

### In-feed GOS modulates host immune response.

**FIG 4:**
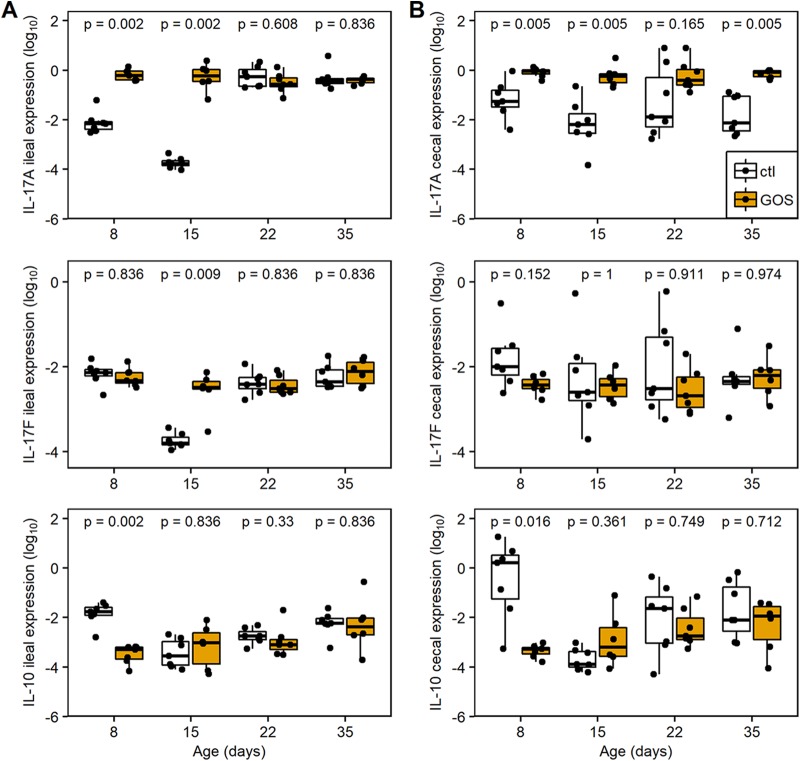
Changes in expression of IL-17A, IL-17F, and IL-10 from ileal (A) and cecal (B) tissues. Relative gene expression was determined by quantitative RT-PCR from total RNAs extracted from cecal tissues for GOS-fed birds (gold) and compared to the expression from birds on the control diet (black). Expression of the gene of interest (GOI) relative to the housekeeping gene (HG) is presented as box-and-whisker plots of data from 7 independent birds determined from 3 technical replicates. The GOS-fed birds analyzed were reduced to 6 for the 15- and 35-da time points due to qPCR data not meeting quality thresholds. The housekeeping genes were the GAPDH and RPL4 genes. The data are recorded as log_10_-transformed 2^−Δ^*^CT^* values. The Δ*C_T_* was calculated (*C_T_* GOI − *C_T_* HG) for each sample; significant differences between 2^−Δ^*^CT^* values of the control and GOS diet cohorts of birds are indicated by *P* values above the sample pairs calculated using the nonparametric Wilcoxon rank sum test with Benjamini-Hochberg false-discovery rate correction.

The immune responses were assessed in ileal and cecal tissues of trial 1 by quantification of the relative expressions of cytokine and chemokine genes representing the major inflammatory pathways of chickens (35). The relative expressions of the anti-inflammatory cytokine interleukin-10 (IL-10) and proinflammatory Th17-associated cytokine IL-17A were profoundly modulated in juvenile birds in both ileal and cecal tissues (Fig. 4; Table 3). In ileal tissues from GOS-fed birds, cytokine expression was marked by upregulation of IL-17A (fold change [FC] = 83; corresponding probability [*Padj*] = 0.002) at 8 da (Fig. 4A; Table 3) and downregulation of IL-10 (FC = 0.02; *Padj *= 0.002), while IL-17F remained unchanged (*Padj *= 0.836). At 15 da, the relative upregulation of ileal IL-17A (FC = 2841; *Padj *= 0.002) and IL-17F (FC = 19; *Padj *= 0.009) was recorded as a consequence of a reduction in expression in the control birds, while expression in the GOS-fed birds remained similar to that at 8 da (Fig. 4B). In cecal tissues the GOS-fed birds were also marked by downregulation of IL-10 (FC = 0.0001; *Padj *= 0.016) at 8 da and upregulation of IL-17A at 8, 15, and 35 da (FC ≥ 12; *Padj *= 0.005).

**TABLE 3:** Modulation of intestinal cytokine and chemokine responses to dietary GOS in broiler chickens^*a*^

Cytokine or chemokine	Ileum								Cecum							
	8 da		15 da		22 da		35 da		8 da		15 da		22 da		35 da	
	FC	*Padj*	FC	*Padj*	FC	*Padj*	FC	*Padj*	FC	*Padj*	FC	*Padj*	FC	*Padj*	FC	*Padj*
IFN-γ	0.5	0.8	1	0.8	1	0.8	3	0.8	0.5	0.6	7	0.2	2	0.9	1	0.9
IL-1β	0.6	0.7	0.3	0.5	0.8	0.9	5	0.5	0.6	0.9	2	0.9	0.4	0.9	3	0.9
IL-4	0.1	0.2	0.1	0.05	2	0.9	3	0.2	1	0.9	5	0.9	2.0	0.9	1	0.9
IL-10	0.02	0.002	2	0.8	0.6	0.3	2	0.8	0.001	0.02	6	0.4	0.5	0.7	0.4	0.7
IL-6	2	0.7	1	0.7	0.3	0.8	0.3	0.2	0.04	0.5	13	0.1	0.3	0.5	1	0.6
ChCXCLi-1	0.7	0.8	2	0.4	16	0.03	2	0.4	0.9	0.9	1	0.9	0.05	0.07	1	0.9
ChCXCLi-2	2	0.4	4	0.2	15	0.1	2	0.4	1	0.7	3	0.5	0.1	0.1	1	0.5
IL-17A	83	0.002	2841	0.002	0.6	0.6	0.6	0.8	12	0.005	88	0.005	11	0.1	60	0.005
IL-17F	0.8	0.8	19	0.009	0.8	0.8	1.1	0.8	0.2	0.1	0.7	1	0.3	0.9	1	0.9

a Cytokine and chemokine gene expression is recorded as fold change (FC) for GOS-fed birds relative to the control diet birds at the same age calculated as 2^−ΔΔ^*^CT^*. Corresponding probabilities (*Padj*) were calculated from log_10_-transformed 2^−Δ^*^CT^* values using nonparametric Wilcoxon rank sum tests adjusted with Benjamini-Hochberg false-discovery rate correction.

At 22 da in GOS-fed birds, proinflammatory chemokine ChCXCLi-1 was increased in the ileum (FC = 16; *Padj *= 0.03) and, conversely, reduced in the ceca (FC = 0.05; *Padj *= 0.07). These observations indicate that the GOS diet or concomitant shifts in the gut microbiota do not drive induction of proinflammatory responses such as IL-1β or Th1-associated gamma interferon (IFN-γ) cytokines, while the reduction of IL-4, a marker for the Th2 pathway, was transient and limited to the ileum at 15 da (FC = 0.1; *Padj *= 0.05).

**FIG 5:**
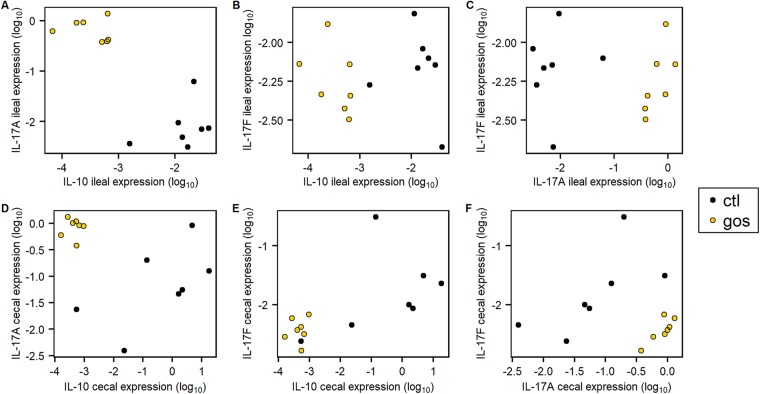
Differential cytokine gene expression in juvenile cecal and ileal tissues. Shown is juvenile IL-17A, IL-17F, and IL-10 differential gene expression relative to those of GAPDH and RPL4 at 8 da in ileal (A, B, and C) and cecal (D, E, and F) tissues of birds on the GOS diet compared to those on the control diet. The 2^−Δ^*^CT^* was determined as indicated in the legend to Fig. 4, and the log_10_-transformed expression values were plotted against each other (*n *= 7).

Figure 5 shows the relative expressions of the IL-17A, IL-17F, and IL-10 genes in the ileum and ceca of 8-da chicks on GOS or control feed. Birds on the GOS and control diets were clearly differentiated on the basis of these data and demonstrated a reduction in the variation in transcription of these specific cytokines in the ceca of birds provided with GOS feed. The increase of IL-17A transcription in the ileal tissues of GOS-fed birds at 8 da coincides with reduced expression of IL-10 (Fig. 5A), whereas IL-17F transcripts were unaffected (Fig. 5B and C). Similarly, the in-feed inclusion of GOS resulted in higher levels of IL-17A and reduced levels of IL-10 transcription in cecal tissues (Fig. 5D) without modulation of IL-17F transcription (Fig. 5E and F). These data suggest differential regulation of IL-17A and IL-17F and that IL-17A responds to in-feed GOS as a component of the Th17 immune response in the ceca, which coincides with the greater relative abundance of L. johnsonii (OTU0010).

### Abundance of L. johnsonii in the cecal lumen positively correlates with bird growth performance.

**FIG 6:**
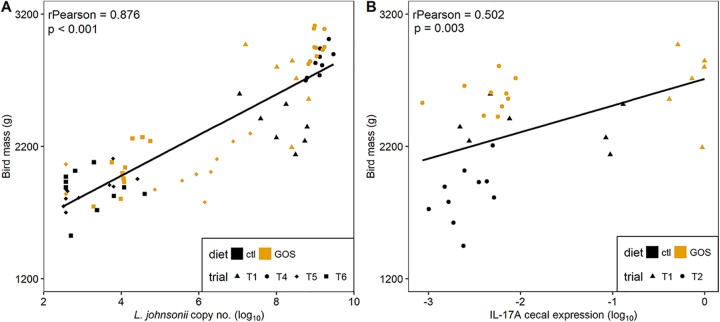
Correlation of growth performance to L. johnsonii abundance and IL-17A expression. Panel A shows the Pearson correlation of bird mass at 35 da against log_10_ L. johnsonii gene copy number per gram of cecal contents determined by quantitative PCR from DNA extracted from cecal contents. Panel B shows the Pearson correlation of the bird weights at 35 da with the cecal IL-17A transcription levels expressed as log_10_-transformed 2^−Δ^*^CT^* values. Gene expression was determined by RT-qPCR from total RNAs extracted from cecal tissues.

Correlations between abundances of the *Lactobacillus* isolates with bird weight were analyzed by combining all the data for 35-da birds from trials 1, 4, 5, and 6, which represent a range of performance outcomes. These data show a clear positive relationship between body mass and the L. johnsonii genome copy number determined by quantitative PCR (qPCR) from the cecal microbiota (Pearson’s *r* = 0.876; *P* ≤ 0.001 [Fig. 6A]). A significant negative correlation between L. crispatus genome copy number and mass was also observed (Pearson’s *r* = −0.763; *P* ≤ 0.001 [data not shown]). The abundances of L. crispatus and L. johnsonii were further analyzed for any relationship with the expression of IL-17A, IL-17F, and IL-10 that we observed to exhibit differential expression on the GOS diet. Figure 6B shows the correlation noted with the expression of IL-17A and L. johnsonii genome copy number (Pearson’s *r* = 0.502; *P = *0.003). These results together strongly suggest that L. johnsonii acts as a key species promoted by GOS to improve growth performance and prime a Th17 immune response.

### Modulation of cecal lactobacilli and bird growth performance.

**FIG 7:**
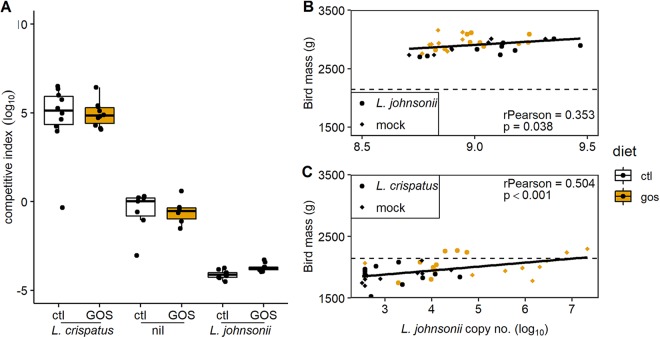
Modulation of the juvenile cecal microbiota. Panel A shows the competitive indices (L. crispatus/L. johnsonii ratios) at 35 da of broiler chickens either nontreated or administered 8 log_10_ CFU of either L. crispatus DC21.1 or L. johnsonii DC22.2 by cloacal gavage at 6 da. Competitive indices were calculated as the ratios of L. crispatus (OTU0006) to L. johnsonii (OTU0010) genome copy numbers per gram of cecal content determined by qPCR. Panel B shows the Pearson correlation of bird mass at 35 da against L. johnsonii gene copy number per gram of cecal content in birds administered L. johnsonii. Panel C shows the Pearson correlation of bird mass at 35 da against L. johnsonii gene copy number per gram of cecal content in birds administered L. crispatus. The dashed horizontal line represents the contemporary male Ross 308 performance objective (96).

To examine if shifts in the cecal abundance of lactobacilli of juvenile broiler chickens can modify growth performance, we administered either L. crispatus DC21.1 or L. johnsonii DC22.2 (8 log_10_ CFU) by cloacal gavage to chicks at 6 da. Cloacal gavage has the advantage of allowing cecal colonization without the impact of upper intestinal transit and accompanying losses in the effective dose of the colonizing bacteria (36). Figure 7A shows marked shifts in the cecal abundance ratios of L. crispatus/L. johnsonii (competitive indices calculated as the ratios of the genome copy numbers per gram of cecal content) at 35 da in favor of the *Lactobacillus* spp. administered compared to the nontreated controls for birds on control or GOS diets. Extreme differences in the cecal abundance of L. johnsonii corresponded with disparate differences in the weights of mature birds at 35 da. The mean body weight of the birds with low cecal L. johnsonii abundance (administered with competitive L. crispatus) fed on the control diet was 1.86 ± 0.16 kg, compared with 2.83 ± 0.11 kg for the birds with high cecal L. johnsonii abundance (*P < *0.001). Figure 7B and C show the respective correlations between body mass and L. johnsonii genome copy number from the cecal microbiota of birds administered either L. johnsonii DC22.2 (Pearson’s *r* = 0.353; *P = *0.038) or L. crispatus DC21.1 (Pearson’s *r* = 0.504; *P < *0.001). These data provide further evidence for the positive relationship between the cecal abundance of L. johnsonii and growth performance. The impact of the early exogenous introduction of L. crispatus DC21.1 was to reduce the abundance of L. johnsonii, which coincided with a reduction in the mean body mass of the birds at 35 da. Although the provision of in-feed GOS under these circumstances led to an increase in the relative abundance of cecal L. johnsonii and improved body masses at 35 da, the birds in the L. crispatus treatment groups did not develop a comparable relative abundance of L. johnsonii or achieve the weight at 35 da observed for mock-treated birds (Fig. 7C). Collectively, these data indicate L. crispatus is a competitor of L. johnsonii that affects differences in the compositional development of the cecal microbiota. Early shifts in the juvenile microbiota have a profound effect on the weights of market-ready broiler chickens. Figure 7C shows that the impact of dietary GOS is greatest on the weaker-performing birds administered L. crispatus, with increased body weight and increased cecal L. johnsonii abundance relative to those on the control diet due to expansion of the niche available to the resident bacteria.

## DISCUSSION

Improvements in the growth performance together with improved health are key goals in broiler chicken production. Studies of broiler chickens aimed to establish productive intestinal microbiota have highlighted compositional shifts in the microbiota that discriminate between birds with opposing zootechnical parameters, but these can vary between experimental trials (15). Several families of bacteria have been reported from the intestinal communities of chickens to show positive or negative associations with feed efficiency: *Bacteroides*, *Enterobacteriaceae*, *Clostridium*, *Ruminococcus*, *Faecalibacterium*, and *Lactobacillus* (15, 37–39).

The inclusion of galacto-oligosaccharides in broiler feed resulted in an enhanced growth rate relative to those of chickens on carefully matched control diets reared under identical conditions. Prebiotic galacto-oligosaccharides have previously been reported to improve the performance and intestinal architecture and stimulate intestinal defenses of neonatal pigs (40). However, a GOS-supplemented diet fed to chickens increased fecal populations of bifidobacteria and lactobacilli but did not improve zootechnical performance (19). In contrast, we observed a significant improvement in performance of the GOS-fed broiler chickens that was also accompanied by changes in the intestinal microbiota. Differences in the abundance of specific members of the *Lactobacillus* genus in the cecal microbiota of juvenile birds on control and GOS diets were observed. *Lactobacillus* isolates were recovered from the cecal contents of these juvenile birds. Among these, specific isolates were identified that shared sequence identity with the OTUs displaying differential abundance in the microbiota of birds consuming either the GOS or the control diet. Whole-genome sequence alignments allowed the identification of L. johnsonii isolate DC22.2, prevalent in the ceca of GOS-fed birds, and L. crispatus isolate DC21.1, exhibiting greater abundance in control birds.

Lactobacilli need to import GOS (degree of polymerization of 2 to 6) and lactose since the enzymes required to break down the substrate are generally cell bound. Two principal pathways accomplish import: LacS lactose permease or the LacE/F phosphotransferase system, where the latter appears to be restricted to DP2 lactose (34). The dependence of LacS for the utilization of GOS with a DP of >2 was first established in L. acidophilus (41). Intestinal *Lactobacillus* species that form the “acidophilus complex” include L. crispatus, L. johnsonii, and L. helveticus, which show conservation of the *gal*-*lac* clusters (41). While the *lacS* gene is often present, the copresence of *lacS* and *lacA* (encoding β-galactosidase) appears to be associated with the ability to utilize GOS. L. johnsonii isolates exhibit host-specific differences, where human and porcine sources frequently possess the *lacS* and *lacA* genes but the poultry isolate FI9785 appears to be deficient due to genome rearrangements (42, 43). However, isolate FI9785 does contain orthologues of *lacE*/*F*. Converse to this, L. johnsonii DC22.2 has retained *lacS* and *lacA* but has lost the *lacE*/*F* genes. The L. johnsonii DC22.2 *lacS* permease gene contains a stop codon that would require initiation from an internal AUG with the loss of the first 31 amino acids based on comparisons with database homologues. However, notable exceptions to this are L. pasteuri and L. gallinarum, which, respectively, initiate translation at the corresponding position or 13 codons downstream. The syntenic position that harbors the lactose phosphotransferase-encoding genes in the L. johnsonii poultry isolate FI9785 (42) features a deletion in L. johnsonii DC22.2 that preserves the *lacR* gene encoding the repressor but dispenses with all the functional components. Paradoxically, in our experiments L. johnsonii DC22.2 did not efficiently utilize GOS *in vitro* but represented a greater differentially abundant component of the cecal microbiota of GOS-fed birds, which exhibited improved zootechnical performance. In contrast, L. crispatus DC21.1 can utilize GOS, but this did not provide a competitive advantage in GOS-fed chickens; rather, the reverse appears to be true. It seems unlikely that L. johnsonii DC22.2 can compete for the GOS substrate directly with L. crispatus DC21.1, which suggests that L. johnsonii DC22.2 benefits from the metabolic capability of another member of the cecal microbiota. The presence of GOS provides the trophic selection for members of the cecal community required to support autochthonous L. johnsonii. Indeed, a hallmark of the acidophilus complex gene contents is the absence of the biosynthetic pathways necessary to produce essential nutrients such as amino acids, purine nucleotides, and cofactors and therefore a reliance on effectively importing nutrients generated by the intestinal milieu (44, 45). Prebiotic selection *in situ* may well be a more reliable way of achieving a beneficial microbiota than directly providing dietary probiotics, as the response will be congruent with the metabolic capabilities of the resident community. Prevailing environmental conditions may alter the content and composition of the intestinal microbiota and therefore the outcomes of prebiotic selection. For example, members of the *Bifidobacterium*, *Christensenella*, and *Lactobacillus* genera have been reported to feature in the intestinal communities of chickens on GOS diets (19, 46, 47). However, removing GOS from the diet at 24 da and feeding the control finisher diet did not alter the performance improvement associated with feeding GOS to the juvenile birds or the succession of L. johnsonii in the process. This would suggest that the impact of juvenile prebiotic feed is lifelong. Recently, Slawinska et al. (48) have reported the delivery of galacto-oligosaccharides *in ovo*, which resulted in an increase in the relative abundance of *Bifidobacterium*-specific 16S rRNA PCR-amplifiable sequences from the cecal contents of mature birds at 42 da, implying that the composition of the mature microbiota can be programmed in early development.

The route to establishing a beneficial microbiota notwithstanding, we observed an increase in the abundance of L. johnsonii in GOS-fed birds that correlated with improved performance. L. johnsonii is an established probiotic with a variety of reported effects when administered to humans and animals. For example, L. johnsonii isolate N6.2 has been shown to have immunomodulatory effects in animal experiments and to protect diabetes-prone rats from developing the disease (49, 50). In another study, L. johnsonii was shown to attenuate respiratory viral infection via metabolic reprogramming and immune cell modulation (51). L. johnsonii LB1 expresses a bile salt hydrolase active against tauro-beta-muricholic acid (T-β-MCA), a critical mediator of farnesoid X receptor (FXR) signaling that is important in maintaining metabolic homeostasis (52). In broiler chickens, administration of an L. johnsonii isolate has been reported to improve growth performance (53). Subsequently, it was reported that meat from L. johnsonii-treated birds had higher nutritional value and the birds showed resistance to the development of necrotic enteritis (54, 55). Similar results were obtained by administering L. johnsonii LB1 to piglets to improve performance and reduce diarrhea (56). The administration of L. johnsonii FI9785 to broiler chickens has also been reported to reduce colonization by Clostridium perfringens, Escherichia coli O78:K80, and Campylobacter jejuni, which have a significant impact on poultry production (57, 58). Lactobacillus crispatus is also a recognized probiotic but is better known for its activity against recurrent urinary infections, bacterial vaginosis, and vaginal candidiasis (59). However, it should be noted that L. crispatus is commonly reported as a major constituent of the chicken microbiome (46, 60).

Shifts in the *Lactobacillus* spp. in response to GOS are accompanied by changes in the expression of cytokines and chemokines that have the potential to prime innate intestinal immune systems and enhance pathogen resistance. However, unchecked low-grade proinflammatory responses can cause tissue damage and inefficient feed conversion (61). Lactic acid, for example, is a by-product of glycolytic pathway of immune cells that can affect local T cell immunity by inhibiting T cell motility and inducing the change of CD4^+^ cells to a Th17 proinflammatory T cell subset, which leads to IL-17 production and chronic inflammation (62). However, lactic acid is also the homofermentative product of lactic acid bacteria such as L. johnsonii, the action of which in the gut has recently been reported to promote the expansion of intestinal stem cells, Paneth cells, and goblet cells (63). Coincident with the increased abundance of L. johnsonii, we observed significantly greater ileal goblet cell densities from juvenile birds on the GOS diet. We also observed an increase in IL-17A and a decrease in IL-10 gene expression in juvenile birds on the GOS diet. IL-17A has been proposed to promote the maintenance of intestinal epithelial cell integrity based on observations that IL-17A inhibition exacerbates colitis in a mouse model, which leads to weakening of the intestinal epithelial barrier (64). In contrast, IL-17F knockout mice are reported to be protected against chemically induced colitis, whereas IL-17A knockout mice remain sensitive (65). Moreover, IL-17F-deficient mice showed an increase in the colonic abundance of *Clostridium* cluster XIVa organisms that promote beneficial regulatory T cells and the expression of β-defensins 1 and 4 (65). Extrapolating from these data, we propose that the increased expression of IL-17A we observed without impact on IL-17F in GOS-fed chickens will promote gut health, a prerequisite for improved commercial production.

The induction of host Th17 responses in ileal and cecal tissues by an indigenous symbiont is reminiscent of the Th17 stimulation brought about by adherent segmented filamentous bacteria (SFB) described for mice (66–68). The tight adherence of SFB to epithelial cells was observed to accelerate postnatal maturation of intestinal mucosal immunity by triggering a Th17 response (69). The observation of an intestinal Th17 response to tightly adherent symbiotes was extended to the human symbiont Bifidobacterium adolescentis in mice (70). However, the response arose through transcriptional program distinctly different from that observed for SFB, suggesting that intestinal Th17 responses are maintained by parallel sensor and signaling pathways. The upregulation of IL-17A in juvenile chickens fed a GOS diet may also support gut immune maturation through Th17 cell stimulation. In chickens, lactobacilli are recognized as adherent to the intestinal tract epithelium from crop to ceca. In addition to established cell adhesion factors, such as the expression of a fibronectin binding protein, it is proposed that L. johnsonii co-opts alternative cell surface-associated structures to cell adhesion roles; these include GroEL, elongation factor Tu, and lipoteichoic acid (71–74).

Juvenile chickens have been reported to exhibit a transient IL-17 induction during the development of the natural microbiota (75). The study suggested that in the absence of IL-22, proinflammatory Th17 induction did not result in intestinal tissue damage. It is possible that IL-17 has a role in the codevelopment of the microbiota and innate immunity in chickens, which is consistent with our findings that upregulation of IL-17A did not cause lamina propria inflammation. Crhanova et al. (75) also reported the outcome of Salmonella enterica serovar Enteritidis challenge of chickens shifts from a Th1 response (induction of IFN-γ and nitric oxide synthase) at 1 to 4 da to a Th17 response at 16 da (induction of IL-17). They conclude that a mature Th17 subset of helper T cells produced IL-17 and IL-22, which confer resistance to *S.* Enteritidis infection and damage in older birds.

### Conclusion.

We have demonstrated an increase in growth rate of broiler chickens in response to dietary supplementation with the prebiotic GOS. Juvenile chickens on GOS starter feed exhibited differences in the cecal abundance of key species of *Lactobacillus* compared to those on control feed. Differences in the cecal microbiota in early development correlated with the composition of the mature cecal microbiota and performance outcomes. The provision of dietary GOS increased the density of goblet cells populating ileal villi in the developing chicken gut. Goblet cell increases were accompanied by significant differences in the villus height and crypt depth at 15 da, a period when transitions in the development of the chicken microbiota from a juvenile to a mature composition are observed (76–78). We have demonstrated a significant correlation between the market weight of chickens at 35 da and the cecal abundance of a specific L. johnsonii isolate identified as differentially abundant in the juvenile microbiota of GOS-fed birds. L. johnsonii abundance also shows a positive correlation with IL-17A gene expression. L. johnsonii is an established probiotic that has been demonstrated to have beneficial effects when applied in poultry production (53–55, 57, 58). Several modes of action have been proposed for probiotic strains of L. johnsonii, but underlying these is the multimodal ability of the species to affect epithelial gut cell adherence (71–74), which we propose will induce the expression of IL-17A. By taking a system-wide approach we have, for the first time, established mechanistic links between prebiotic selection of an autochthonous synbiotic species, increased IL-17A expression, and the development of the gut in healthy animals.

## MATERIALS AND METHODS

### Ethical approval.

Experiments involving the use of birds were subjected to an approval process under national guidelines by the United Kingdom Home Office. Work on this project was approved under United Kingdom Government Home Office project licensing ASPA 86. All project licenses are reviewed internally by the University Ethics Committee prior to submission to the Home Office. This includes the scrutiny of animal welfare, ethics, and handling.

### Experimental birds.

Commercial male Ross 308 broiler chicks were obtained as hatchlings (PD Hook, Oxfordshire, UK). Birds were housed in a controlled environment under strict conditions of biosecurity. Temperatures were as outlined in the *Code of Practice for the Housing and Care of Animals Bred, Supplied or Used for Scientific Purposes* (79). Birds were provided feed and water *ad libitum*. Feeds were formulated on a least-cost basis and to meet the requirements set out in the *Ross 308 Broiler Nutrition Specifications 2014* (80) and prepared by Target Feeds Ltd (Shropshire, UK). The diet regime was as follows: the control diet group was sustained on a wheat-based diet provided as a starter crumb for 0 to 10 days of age (da), grower pellets for 11 to 24 da, and finisher pellets for 25 to 35 da. The starter diet contained wheat (59.9% [wt/wt]), soya meal (32.5% [wt/wt]), soyabean oil (3.65% [wt/wt]), limestone (0.6% [wt/wt]), calcium phosphate (1.59% [wt/wt]), sodium bicarbonate (0.27% [wt/wt]), the enzymes phytase and xylanase (dosed according to the manufacturer’s instructions; DSM Nutritional Products Ltd., Basel, Switzerland), and a vitamin mix containing salt, lysine hydrochloride, dl-methionine, and threonine. The grower and finisher diets increased the wheat content at the expense of soya meal by 2 and 5% (wt/wt), respectively. GOS was provided as Nutrabiotic (GOS, 74% [wt/wt] dry matter) (Dairy Crest Ltd., Davidstow, Cornwall, UK). Galacto-oligosaccharide preparations contained a mixture of monosaccharides (glucose and galactose) and oligosaccharides (DP2 to DP8). The disaccharide lactose, a reactant in the manufacture of galacto-oligosaccharides, is not a galacto-oligosaccharide; all other disaccharides and longer oligosaccharides (DP3+) are considered to be galacto-oligosaccharides and nondigestible. The starter feed was supplemented with 3.37% (wt/wt) GOS and isocaloric adjustments made in the wheat (54% [wt/wt]) and soybean oil (4.88% [wt/wt]) contents. The grower and finisher feeds contained 1.685% GOS with respective adjusted wheat contents of 57.7% (wt/wt) and 63.3% (wt/wt) and soybean oil contents of 6.14% (wt/wt) and 6.22% (wt/wt). The final feeds were isocaloric (metabolizable energy including enzyme contribution) and contained the same crude protein levels and Degussa poultry digestible amino acid values (lysine, methionine, methionine plus cysteine, threonine, tryptophan, isoleucine, valine, histidine, and arginine). The feed formulations are listed in Table S2.

10.1128/mSystems.00827-19.4 TABLE S2 GOS and control feed formulations. Feed formulations for starter (0 to 10/11 days of age), grower (11/12 to 24 days of age), and finisher (25 to 35 days of age) diets are shown. Download Table S2, DOCX file, 0.02 MB. Copyright © 2020 Richards et al. 2020 Richards et al. This content is distributed under the terms of the Creative Commons Attribution 4.0 International license.

Chickens were euthanized by either exposure to rising CO_2_ gas or parenteral barbiturate overdose followed by cervical dislocation according to schedule 1 of the UK Animals (Scientific Procedures) Act. The birds were weighed before tissue and intestinal contents were sampled postmortem. Ileal tissues were sectioned from approximately 3 cm distal to Meckel’s diverticulum, and cecal tissues were collected from the distal tips of the ceca. Intestinal tissues were immediately frozen in liquid nitrogen for subsequent RNA isolation or preserved in 10% (wt/vol) neutral buffered formalin (Fisher Scientific, Loughborough, UK) for histological assessment. Intestinal contents were collected and stored at –80°C until DNA isolation.

### Trial designs. (i) GOS diet trial 1.

On the day of hatch, chicks were randomly assigned to either a control diet or a GOS-supplemented diet for the duration of the experiment. Two groups of 35 birds were kept in pens from day of hatch until day 6, when all birds were caged independently until euthanasia of 7 birds at sample time points 8, 15, and 22 days of age (da) to obtain intestinal contents and tissue biopsy specimens. Birds were weighed and feed consumption was recorded at least weekly from the start of the experiment at the day of hatch until then end of the study at 35 da. Growth rates between 8 and 35 da were determined for 10 birds remaining at the end of trial and for which feed consumption and live weights were recorded over the entire trial period. Feed conversion ratios (FCR) were calculated as a ratio of the cumulative feed consumed to the weights of the birds.

### (ii) Ancillary GOS diet trials 2 and 3.

To advance the study, we modified the experimental design to establish if removing the prebiotic feed after the microbiota had been established in the juvenile birds could reproduce the beneficial effects on performance observed for mature birds. These studies used the starter and grower feed formulations listed in Table S3, but both GOS treatment and control birds in ancillary trial 2 and ancillary trial 3 were fed the control finisher diet 25 to 35 da. The organization of trial 2 was the same as that of trial 1 (*n *= 10), while for trial 3, the birds were cohoused on wood shavings in 10 pens of 3 birds and wing tagged to identify individual birds instead of individual caging. Feed consumption was measured per pen and calculated as the average per bird for each pen (*n *= 10).

10.1128/mSystems.00827-19.5 TABLE S3 Primer sequences used for PCR. Download Table S3, DOCX file, 0.02 MB. Copyright © 2020 Richards et al. 2020 Richards et al. This content is distributed under the terms of the Creative Commons Attribution 4.0 International license.

### (iii) Cloacal gavage trials 4, 5, and 6.

On the day of hatch, chicks were randomly assigned to either the control or GOS-supplemented diet. At 6 da, axenic suspensions of either L. crispatus DC21.1 or L. johnsonii DC22.2 containing between 7.4 and 7.8 log_10_ CFU in 0.1 ml of MRD (Oxoid, Basingstoke, UK) were administered to chicks by cloacal gavage. Control groups were mock administered with MRD alone. Cloacal gavage was performed using a blunt narrow-nosed syringe to stimulate reverse peristalsis. Postgavage, experimental groups of 20 birds were housed in pairs and maintained on GOS or control diet (*n *= 10). The birds were fed either the control diet throughout or the GOS diet until 24 da and then switched to the control until the end of the trial at 35 da. Feed consumption was measured per pen and calculated as the average per bird for each pen.

### Identification of lactic acid bacteria.

Cecal contents from each individual bird were serially diluted in MRD (Oxoid) and spread (0.1 ml) onto the surfaces of MRS (Oxoid) plates. The MRS plates were incubated under anaerobic conditions for 48 h at 37°C. The numbers of lactic acid bacterial colonies were recorded, and examples of distinct, well-isolated colonies were subcultured for identification and storage at –80°C. Multiple isolates from MRS plates were examined by microscopy using the Gram stain. Genomic DNAs were prepared from selected isolates showing different cell and colony morphologies using a GenElute bacterial genomic DNA kit (Sigma Aldridge, Gillingham, UK). Identification to presumptive species level was carried out by performing PCR amplification of 16S rRNA gene sequences using the primers 27f and 1522r (Table S3) (81, 82) and DNA sequencing of the products following cleanup (Wizard SV gel and PCR cleanup system; Promega, Southampton, Hampshire, UK) using dye terminator chemistry (Eurofins, Ebersberg, Germany). The 16S rRNA gene V4 region sequences were matched to the OTU clusters outputted from microbiome analysis. The genome sequences of L. crispatus DC21.1 and L. johnsonii DC22.2 were assembled using CLC Genomics Workbench 10.0.1 (Qiagen, Aarhus, Denmark) using a combination of data generated from the Illumina MiSeq and PacBio RSII platforms. The L. crispatus DC21.1 and L. johnsonii DC22.2 cultures were deposited at the National Collection of Industrial Food and Marine Bacteria (NCIMB) under the respective accession numbers 42771 and 42772.

### *In vitro* growth of *Lactobacillus* on galacto-oligosaccharides.

To determine the ability of L. crispatus DC21.1 and L. johnsonii DC22.2 to utilize GOS *in vitro*, a purified Nutrabiotic GOS (74% [wt/wt] dry matter) containing a DP2+ lactose fraction was prepared using high-performance liquid chromatography (HPLC), with an Imtakt Unison UK-Amino (aminopropyl stationary phase) column (ARC Sciences, Oakham, UK) with acetonitrile-water mobile phase, to remove monomeric sugars (glucose and galactose) and lactose (IPOS Ltd., Huddersfield, UK). A reduced-carbon-source medium based on MRS broth with the omission of glucose was prepared as a basal medium (pH 6.7). One liter of the medium contained 10 g of tryptone (Oxoid), 5 g of yeast extract (Oxoid), 10 g of Lab-Lemco powder (Oxoid), 1 ml of sorbitan mono-oleate (Tween 80), 2 g of dipotassium hydrogen phosphate, 0.5 g of sodium acetate 3H_2_O, 2 g of diammonium hydrogen citrate, 0.2 g of magnesium sulfate 7H_2_O, and 0.05 g of manganese sulfate 4H_2_O (from Fisher Scientific unless otherwise stated). Each experiment was carried out using the basal medium with addition of DP2+ lactose GOS (0.5% [wt/vol]), together with a positive control, containing glucose (0.5% [wt/vol]) and a negative control with sterile water instead of the carbon source. The bacterial cultures were grown on MRS plates and suspended in the modified MRS medium to a density of 8 log_10_ CFU/ml (OD_600_ of approximately 1.5). The suspension was diluted 1 in 100 into the growth medium. The assay was carried out in triplicate with 3 technical replicates per biological replicate together with a set of uninoculated negative controls as blanks (0.2 ml in microtiter plates). The plates were covered and incubated at 37°C for 72 h under anaerobic conditions, with shaking. The OD_600_ obtained from growth on the basal medium, without the addition of a carbon source, was subtracted from the value of the growth on the selected carbon source.

### Histology.

Tissue samples fixed in a 10% formalin solution were dehydrated through a series of alcohol solutions, cleared in xylene, and embedded in paraffin wax (Microtechnical Services Ltd., Exeter, UK). Sections (3 to 5 μm thick) were prepared and stained with either modified hematoxylin and eosin (H&E) or periodic acid-Schiff (PAS) using standard protocols. After staining, the slides were scanned with the NanoZoomer digital pathology system (Hamamatsu, Welwyn Garden City, UK). Measurements of villus height and crypt depth were made using the NanoZoomer digital pathology image program (Hamamatsu). Ten well-oriented villi per tissue section of 4 birds from each diet group at each sampling time were scanned at 40× resolution for each tissue sample. Villus height was measured from the tip of the villus to the crypt opening, and the associated crypt depth was measured from the base of the crypt to the level of the crypt opening. The ratio of villus height to relative crypt depth was calculated from these measurements. Goblet cells were enumerated from ileal sections stained with PAS. In one case the histology section did not fulfill the quality criterion of 10 well-oriented villi and was omitted from the analysis.

### RNA isolation and RT-qPCR of cytokines and chemokines.

RNAs were isolated from cecum and ileum tissue biopsy specimens using a NucleoSpin RNA purification kit (Macherey-Nagel, GmbH & Co. KG, Düren, Germany) according to the manufacturer’s protocol, with the following modifications. Tissue samples were homogenized with the kit lysis buffer and 2.8-mm ceramic beads (MO BIO Laboratories Inc., Carlsbad, CA) using TissueLyser II (Qiagen, Hilden, Germany). Subsequently, total RNA was extracted as described in the protocol with a DNase I treatment step as per the manufacturer’s instructions. Purified RNAs were eluted in nuclease-free water, validated for quality and quantity using UV spectrophotometry (Nanodrop ND-1000; Labtech International Ltd., Uckfield, UK), and stored long term at –80°C. RNAs with OD_260_/OD_280_ ratios between 1.9 and 2.1 were deemed high quality; the sample ratios had a mean of 2.12 ± 0.01. Reverse transcription (RT) was performed with 1 μg of RNA, SuperScript II (Invitrogen Life Technologies, Carlsbad, CA), and random hexamers as described previously (76).

Quantitative PCR was performed with cDNA templates derived from 4 ng of total RNA in triplicate using SYBR green master mix (Applied Biosystems, Thermo Fisher Scientific, UK). The RNA level of expression was determined by qPCR using the Roche Diagnostics LightCycler 480 (Hoffmann La Roche AG, Switzerland). The primer sequences for glyceraldehyde-3-phosphate dehydrogenase (GAPDH), RPL4, IFN-γ, IL-1β, IL-4, IL-6, IL-10, IL-17A, IL-17F, ChCXCLi-1, and ChCXCLi-2 (83–86) are presented in Table S3. Cytokine and chemokine transcript levels and fold change were calculated according to the manufacturer’s recommendation using the 2^−Δ^*^CT^* and 2^−ΔΔ^*^CT^* methods, where the *CT* is the PCR cycle threshold and ∆*CT* corresponds to the difference between the *CT* values of the cyto/chemokine gene and the reference housekeeping genes to give normalized transcript levels, and ∆∆*CT* is the difference between transcript levels of birds on the GOS and control diets (87). The means of triplicate *C_T_* values were used for analysis, where target gene *C_T_* values were normalized to those of the housekeeping GAPDH and 60S ribosomal protein L4 (RPL4) genes.

### Microbiome analysis.

DNA was isolated from cecal contents using the MoBio PowerSoil kit (now Qiagen Ltd., Manchester, UK) according to the manufacturer’s instructions. For microbiome analysis the V4 regions of the bacterial 16S rRNA genes were PCR amplified using the primers 515f and 806r (Table S3) (88). Amplicons were then sequenced on the Illumina MiSeq platform using 2 × 250-bp cycles. The 16S rRNA gene sequences were quality filtered and clustered into OTUs in Mothur (89, 90) using the Schloss lab. MiSeq SOP (https://www.mothur.org/wiki/MiSeq_SOP, accessed 10 May 2018 [91]). Batch files of Mothur commands used in this study are available at https://github.com/PJRichards/Richards_GOS_broiler. Postprocessing rarefaction curves were plotted to assess sampling effort (Fig. S2).

10.1128/mSystems.00827-19.2 FIG S2 Rarefaction curves indicating coverage of cecal bacterial communities. 16S rRNA gene bacterial communities from control diet-fed birds 8 to 35 days of age as indicated (black) (top) and GOS-supplemented-diet-fed birds 8 to 35 days of age as indicated (gold) (bottom). Download FIG S2, TIF file, 2.7 MB. Copyright © 2020 Richards et al. 2020 Richards et al. This content is distributed under the terms of the Creative Commons Attribution 4.0 International license.

### Quantitative PCR enumeration of lactobacilli.

Quantitative PCR protocols to enumerate L. crispatus DC21.1 and L. johnsonii DC22.2 organisms from intestinal contents were developed by designing primers specific for the *groEL* gene sequences of these bacterial strains (Table S3). Real-time qPCR quantification of L. crispatus DC21.1 and L. johnsonii DC22.2 was performed with 1 μl of cecal content DNA (15 to 150 ng) using SYBR green master mix with the Roche Diagnostics LightCycler 480. The amplification conditions were denaturation at 95°C for 5 min followed by 45 cycles of 95°C for 15-s denaturation and 60°C for 1-min annealing. The fluorescence signals were measured at the end of each annealing step. Melting curves were generated by heating the samples from 65°C to 97°C at a ramp rate of 0.11°C per s. The data obtained were plotted against a standard curve generated with 5-fold serially diluted target bacterial DNAs. Genome copy number of target bacteria in each dilution was calculated based on its genome length and applied DNA quantity with the assumption of the mean molecular mass of one base pair as 650 Da. The method was validated first with DNA extracts from pure cultures of known CFU and then by spiking chicken cecal samples with increasing concentrations of target cells. The data were calculated as genome copy number per microliter of DNA applied in the PCR and converted into genome copy number per gram of cecal content based on the mass of cecal material and elution volume applied for DNA extraction.

### Statistical analysis.

Growth rate was tested for significance by measuring the weight gain for each bird between 8 and 35 days and testing the difference between the growth rates (grams per day) using Student’s *t* test. Differences in bird mass at 35 days were compared using Student’s *t* test.

Competitive indices of the *Lactobacillus* species present in the ceca were calculated as the ratio of the L. crispatus (OTU0006) copy number per gram of cecal content to the L. johnsonii (OTU0010) copy number per gram of cecal content determined using qPCR.

For the microbiota β-diversity analysis, Bray-Curtis distances were tested for significance using analysis of molecular variance (AMOVA) implemented within Mothur (89). Linear discriminant analysis effect size (LefSe) (92) was also implemented within Mothur (89). Differences in α-diversity, Chao richness, and absolute abundance of *Lactobacillus* spp. were tested using Wilcoxon rank sum test. With the exception of Fig. 5, all figures were drawn using R version 3.6.1 (7 May 2019) (93) in Rstudio 1.2 (94). R scripts used to draw figures are available at https://github.com/PJRichards/Richards_GOS_broiler.

Significance tests of heterophil counts and villus and crypt measurements were performed using single-factor analysis of variance (ANOVA) with a *P* value of <0.05 used as the level significance. Non-normally distributed gene expression data were compared using the nonparametric Wilcoxon rank sum tests adjusted with the Benjamini-Hochberg false-discovery rate correction (95).

### Data availability.

The genome DNA sequences of L. crispatus DC21.1 appear in the NCBI database under the accession numbers CP039266 to CP039267. The genome DNA sequences of L. johnsonii DC22.2 appear in the NCBI database under the accession numbers CP039261 to CP039265. Raw DNA sequence data and metadata in support of 16S rRNA metagenomic analysis appear in the NCBI database within Bioproject PRJNA380214. Raw zootechnical observations are available at https://github.com/PJRichards/Richards_GOS_broiler/tree/master/zootechnical.
