# Supplementary material for: Galacto-Oligosaccharides Modulate the Juvenile Gut Microbiome and Innate Immunity To Improve Broiler Chicken Performance
Source: mSystems. 2020 Jan 14;5(1):e00827-19. doi: 10.1128/mSystems.00827-19 (PMC6967391; doi:10.1128/mSystems.00827-19)
Supplement: TABLE S1 [file mSystems.00827-19-st001.docx]

**Supplementary Table S1**

**Ross 308 Broiler chickens fed nutritionally matched control and GOS diets**

| **Diet** | **Control** | **GOS** | **p-value** |
| --- | --- | --- | --- |
| Trial T1 GOS treatment 0-35 da (n=10) | | | |
| Mean body mass at day of hatch (g) | 45.9 | 44.8 | 0.4152118 |
| Mean body mass at day 35 (g) | 2,336 | 2,582 | 0.0409658 |
| Mean BWG 0 - 10 da (g) | 325 | 333 | 0.6632645 |
| Feed per BWG 0 - 10 da | 1.025 | 1.064 |  |
| Mean BWG 11 - 35 da (g) | 1,987 | 2,238 | 0.0383560 |
| Feed per BWG 11 - 35 da | 1.551 | 1.512 |  |
| Cumulative feed intake per bird – 35da (g) | 3452 | 3756 |  |
| FCR (including initial body weight) | 1.478 | 1.455 |  |
| Trial T2 GOS treatment 0-24 da & control 25-35 da (n=10) | | | |
| Mean body mass at day of hatch (g) | 43.7 | 41.2 | 0.1171804 |
| Mean body mass at day 35 (g) | 1,838 | 2,584 | 0.0000001 |
| Mean BWG 0 - 11 da (g) | 284 | 320 | 0.0045978 |
| Feed per BWG 0 - 11 da | 1.075 | 1.056 |  |
| Mean BWG 12 - 35 da (g) | 1,510 | 2,223 | 0.0000001 |
| Feed per BWG 12 - 35 da | 1.647 | 1.433 |  |
| Cumulative feed intake per bird – 35da (g) | 2823 | 3563 |  |
| FCR (including initial body weight) | 1.536 | 1.379 |  |
| Trial T3 GOS treatment 0-24 da & control 25-35 da (n=10) | | | |
| Mean body mass at day of hatch (g) | 38.8 | 38.5 | 0.7792273 |
| Mean body mass at day 35 (g) | 2,291 | 2,501 | 0.0566421 |
| Mean BWG 0 - 10 da (g) | 247 | 262 | 0.0346623 |
| Feed per BWG 0 - 10 da | 0.996 | 0.995 |  |
| Mean BWG 11 - 35 da (g) | 2,044 | 2,239 | 0.0873694 |
| Feed per BWG 11 - 35 da | 1.425 | 1.381 |  |
| Cumulative feed intake per bird – 35da (g) | 3159 | 3354 |  |
| FCR (including initial body weight) | 1.379 | 1.341 |  |
| Trial T4 *L. johnsonii* at 6da, GOS treatment 0-24 da & control 25-35 da (n=10) | | | |
| Mean body mass at day of hatch (g) | 44.6 | 46.0 | 0.1540935 |
| Mean body mass at day 35 (g) | 2,836 | 2,961 | 0.0187776 |
| Mean BWG 0 - 14 da (g) | 404 | 435 | 0.0201485 |
| Feed per BWG 0 - 14 da | 1.203 | 1.112 |  |
| Mean BWG 15 - 35 da (g) | 2,387 | 2,480 | 0.0383560 |
| Feed per BWG 15 - 35 da | 1.411 | 1.398 |  |
| Cumulative feed intake per bird – 35da (g) | 3874 | 3929 |  |
| FCR (including initial body weight) | 1.366 | 1.326 |  |
| Trial T5 mock at 6da, GOS treatment 0-24 da & control 25-35 da (n=10) | | | |
| Mean body mass at day of hatch (g) | 39.5 | 38.6 | 0.2869586 |
| Mean body mass at day 35 (g) | 1,867 | 2014 | 0.0367011 |
| Mean BWG 0 - 11 da (g) | 281 | 287 | 0.4932771 |
| Feed per BWG 0 - 11 da | 1.195 | 1.142 |  |
| Mean BWG 12 - 35 da (g) | 1,565 | 1,640 | 0.3940673 |
| Feed per BWG 12 - 35 da | 1.667 | 1.587 |  |
| Cumulative feed intake per bird – 35da (g) | 2823 | 3563 |  |
| FCR (including initial body weight) | 1.577 | 1.456 |  |
| Trial T6 *L. crispatus* at 6da, GOS treatment 0-24 da & control 25-35 da (n=10) | | | |
| Mean body mass at day of hatch (g) | 38.9 | 38.9 | 0.9719941 |
| Mean body mass at day 35 (g) | 1,869 | 2,032 | 0.0470342 |
| Mean BWG 0 - 10 da (g) | 285 | 290 | 0.6558510 |
| Feed per BWG 0 - 10 da | 1.133 | 1.169 |  |
| Mean BWG 11 - 35 da (g) | 1,598 | 1,723 | 0.1089529 |
| Feed per BWG 11 - 35 da | 1.885 | 1.658 |  |
| Cumulative feed intake per bird – 35da (g) | 2872 | 3195 |  |
| FCR (including initial body weight) | 1.536 | 1.572 |  |

BWG represents body weight gain. FCR represents the feed conversion ratio.
