# Supplementary figures and images for: Galacto-Oligosaccharides Modulate the Juvenile Gut Microbiome and Innate Immunity To Improve Broiler Chicken Performance
Source: mSystems. 2020 Jan 14;5(1):e00827-19. doi: 10.1128/mSystems.00827-19 (PMC6967391; doi:10.1128/mSystems.00827-19)

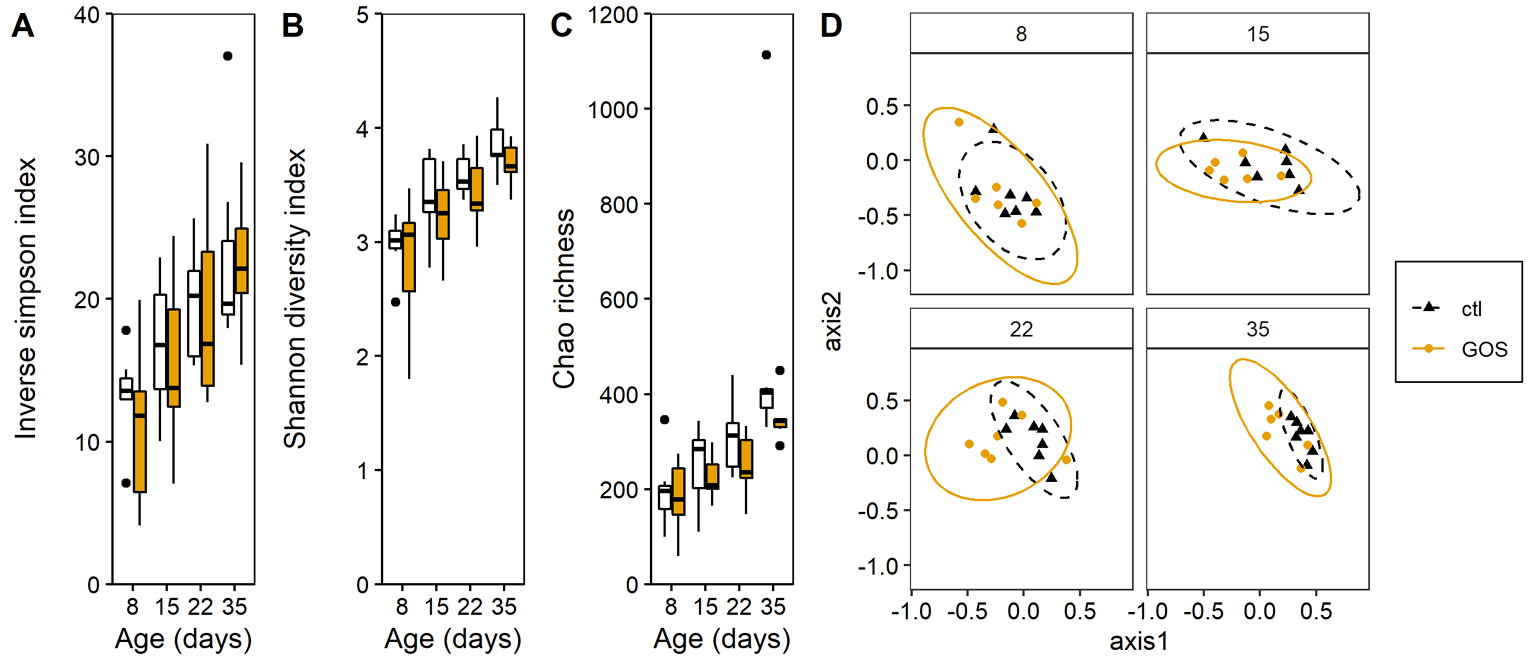

Supplement: FIG S1 [file mSystems.00827-19-sf001.tif]

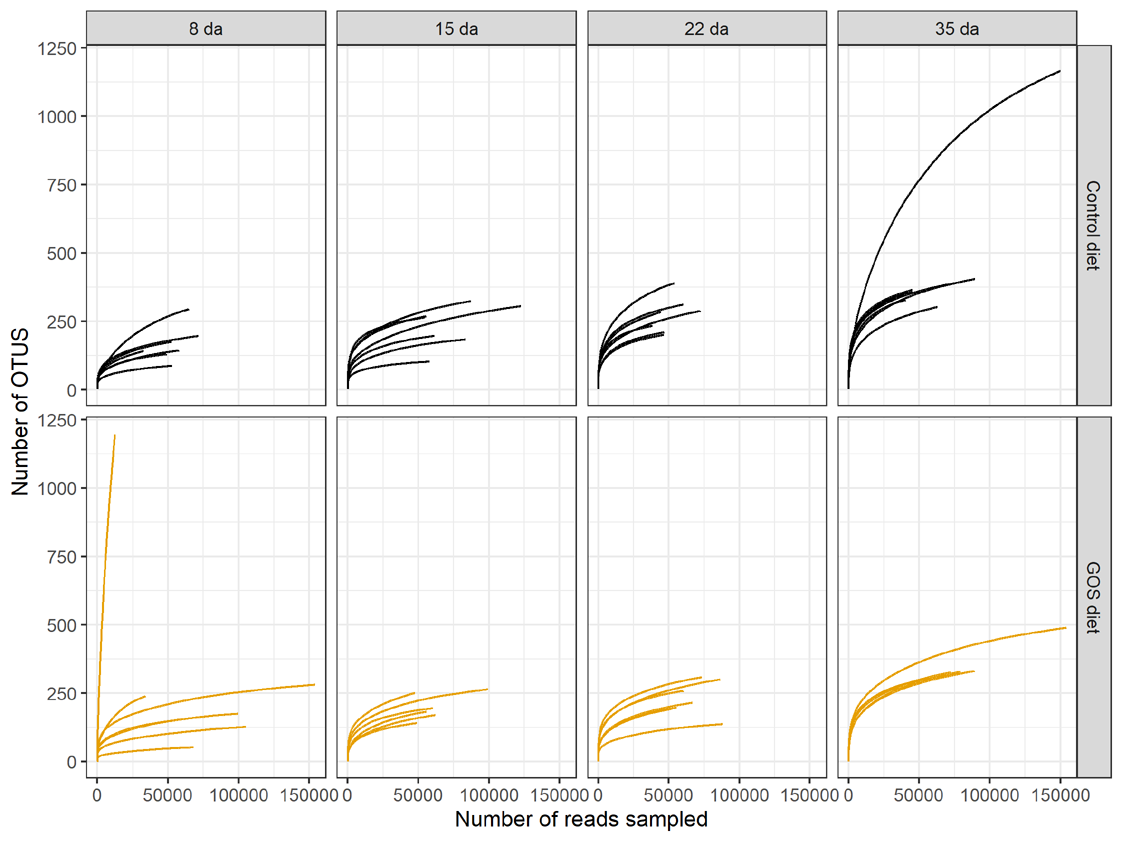

Supplement: FIG S2 [file mSystems.00827-19-sf002.tif]
