# Supplementary material for: Galacto-Oligosaccharides Modulate the Juvenile Gut Microbiome and Innate Immunity To Improve Broiler Chicken Performance
Source: mSystems. 2020 Jan 14;5(1):e00827-19. doi: 10.1128/mSystems.00827-19 (PMC6967391; doi:10.1128/mSystems.00827-19)
Supplement: TABLE S2 [file mSystems.00827-19-st002.docx]

| **FEED COMPONENT** | CONTROL: STARTER | CONTROL:   GROWER | CONTROL:  FINISHER | GOS 3.370%:   STARTER | GOS 1.685%:  GROWER | GOS 1.685%:  FINISHER |
| --- | --- | --- | --- | --- | --- | --- |
| WHEAT | 59.999 | 60.716 | 66.319 | 54.016 | 57.719 | 63.324 |
| EXT. HIPRO SOYA MEAL | 32.5 | 30.8 | 25.3 | 33.9 | 31.5 | 26.0 |
| LIMESTONE GRANULES | 0.60 | 0.40 | 0.40 | 0.60 | 0.40 | 0.40 |
| SOYABEAN OIL | 3.65 | 5.52 | 5.60 | 4.88 | 6.14 | 6.22 |
| LYSINE HCL | 0.296 | 0.119 | 0.123 | 0.264 | 0.103 | 0.107 |
| METHIONINE DL | 0.362 | 0.263 | 0.231 | 0.366 | 0.265 | 0.232 |
| DICALCIUM PHOSPHATE | 1.59 | 1.28 | 1.12 | 1.61 | 1.29 | 1.13 |
| SODIUM BICARBONATE | 0.269 | 0.188 | 0.193 | 0.249 | 0.179 | 0.183 |
| SALT | 0.150 | 0.210 | 0.210 | 0.170 | 0.220 | 0.220 |
| THREONINE | 0.134 | 0.054 | 0.054 | 0.125 | 0.049 | 0.049 |
| TM - Blank Premix for Broiler Formulation | 0.400 | 0.400 | 0.400 | 0.400 | 0.400 | 0.400 |
| NUTRABIOTIC SYRUP (GOS 74% w/w) | **0.000** | **0.000** | **0.000** | **3.370** | **1.685** | **1.685** |
| RONOZYME P5000 (CT) | 0.030 | 0.030 | 0.030 | 0.030 | 0.030 | 0.030 |
| Ronozyme WX (Xyl) | 0.020 | 0.020 | 0.020 | 0.020 | 0.020 | 0.020 |
