# Supplementary material for: Galacto-Oligosaccharides Modulate the Juvenile Gut Microbiome and Innate Immunity To Improve Broiler Chicken Performance
Source: mSystems. 2020 Jan 14;5(1):e00827-19. doi: 10.1128/mSystems.00827-19 (PMC6967391; doi:10.1128/mSystems.00827-19)
Supplement: TABLE S3 [file mSystems.00827-19-st003.docx]

**Supplementary Table 3 Primer sequences used in this study**

| Target gene | Primer sequence (5’-3’) | Product size (bp) | NCBI Accession/name | Reference |
| --- | --- | --- | --- | --- |
| GAPDH | F: GACGTGCAGCAGGAACACTA  R: TCTCCATGGTGGTGA AGACA | 343 | [NM_204305.1](http://www.ncbi.nlm.nih.gov/entrez/viewer.fcgi?db=nucleotide&id=46048960) | 1 |
| INF-γ | F: TGAGCCAGATTGTTTCGATG  R: CTTGGCCAGGTCCATGATA | 152 | NM_205149.1 | 1 |
| IL-1β | F: GGATTCTGAGCACACCACAGT  R: TCTGGTTGATGTCGAAGATGTC | 272 | NM_204524.1 | 1 |
| IL-4 | F: GGAGAGCATCCGGATAGTGA  R: TGACGCATGTTGAGGAAGAG | 186 | NM_001007079.1 | 1 |
| IL-10 | F: GCTGCGCTTCTACACAGATG  R: TCCCGTTCTCATCCATCTTC | 203 | NM_001004414.2 | 1 |
| IL-6 | F: GCTCGCCGGCTTCGA  R: GGTAGGTCTGAAAGGCGAACAG | 71 | [NM_204628.1](http://www.ncbi.nlm.nih.gov/entrez/viewer.fcgi?db=nucleotide&id=45382888) | 2 |
| IL-17A | F: CATGGGATTACAGGATCGATGA  R: GCGGCACTGGGCATCA | 68 | [NM_204460.1](http://www.ncbi.nlm.nih.gov/entrez/viewer.fcgi?db=nucleotide&id=45383240) | 3 |
| IL-17F | F: TGACCCTGCCTCTAGGATGATC  R: GGGTCCTCATCGAGCCTGTA | 78 | [XM_426223.5](http://www.ncbi.nlm.nih.gov/entrez/viewer.fcgi?db=nucleotide&id=971391097) | 3 |
| ChCXCLi-1 | F: CCGATGCCAGTGCATAGAG  R: CCTTGTCCAGAATTGCCTTG | 191 | [NM_205018.1](http://www.ncbi.nlm.nih.gov/entrez/viewer.fcgi?db=nucleotide&id=45384323) | 4 |
| ChCXCLi-2 | F: CCTGGTTTCAGCTGCTCTGT  R: GCGTCAGCTTCACATCTTGA | 128 | [NM_205498.1](http://www.ncbi.nlm.nih.gov/entrez/viewer.fcgi?db=nucleotide&id=49169792) | 4 |
| *L. johnsonii* GroEL | F: GGAAAACATGGTTAAGGCTGGT  R: GAGCTGCAGGAGCTTGGTTC | 152 | E6A54_02330 | Present study |
| *L. crispatus* GroEL  16S rRNA  16S rRNA | F: GAAGGCTATCAATGATCTTAAGG  R: AGCAGAAGAGCGGCAATTGA  F: AGAGTTTGATCMTGGCTCAG  R: AAGGAGGTGATCCANCCRCA  F: GTGCCAGCMGCCGCGGTAA  R: GGACTACHVGGGTWTCTAAT | 276  1557  292 | E6A57_02020  TPU1  RTU8  515f  806r | Present study  5  6 |

1. Nang NT, Lee JS, Song BM, Kang YM, Kim HS, Seo SH. 2011. Induction of inflammatory cytokines and Toll-like receptors in chickens infected with avian H9N2 influenza virus. Vet Res 42:64. doi: 10.1186/1297-9716-42-64.
2. Kaiser P, Staheli P. Avian cytokines and chemokines. In: Schat KA, Kaspers B, Kaiser P, editors. Avian immunology. London: Elsevier; 2014. p. 189–204.
3. Reid WD, Close AJ, Humphrey S, Chaloner G, Lacharme-Lora L, Rothwell L, Kaiser P, Williams NJ, Humphrey TJ, Wigley P, Rushton SP. 2016. Cytokine responses in birds challenged with the human food-borne pathogen Campylobacter jejuni implies a Th17 response. R Soc Open Sci 3:150541. doi: 10.1098/rsos.150541
4. Rasoli M, Yeap SK, Tan SW, Moeini H, Ideris A, Bejo MH, Alitheen NB, Kaiser P, Omar AR. 2014. Alteration in lymphocyte responses, cytokine and chemokine profiles in chickens infected with genotype VII and VIII velogenic Newcastle disease virus. Comp Immunol Microbiol Infect Dis 37:11-21.
5. Ott SJ, Musfeldt M, Ullmann U, Hampe J, Schreiber S. 2004. Quantification of intestinal bacterial populations by real-time PCR with a universal primer set and minor groove binder probes: a global approach to the enteric flora. J Clin Microbiol 42:2566-2572.
6. Caporaso JG, Lauber CL, Walters WA, Berg-Lyons D, Lozupone CA, Turnbaugh PJ, Fierer N, Knight R. 2011. Global patterns of 16S rRNA diversity at a depth of millions of sequences per sample. Proc Natl Acad Sci U S A 108: Suppl 1:4516-22. doi: 10.1073/pnas.1000080107.
